# Molecular evolution and functional divergence of alcohol dehydrogenases in animals, fungi and plants

**DOI:** 10.1590/1678-4685-GMB-2017-0047

**Published:** 2018

**Authors:** Claudia E. Thompson, Loreta B. Freitas, Francisco M. Salzano

**Affiliations:** 1 Departamento de Farmacociências, Unidade de Genômica e Bioinformática Clínica, Universidade Federal de Ciências da Saúde de Porto Alegre, Porto Alegre, RS, Brazil; 2 Unidade de Biologia Teórica e Computacional, Centro de Biotecnologia, Universidade Federal do Rio Grande do Sul, Porto Alegre, RS, Brazil; 3 Departamento de Genética, Instituto de Biociências, Universidade Federal do Rio Grande do Sul, Porto Alegre, RS, Brazil

**Keywords:** Glycolytic proteins, molecular evolution, alcohol dehydrogenase, functional diversification, positive selection

## Abstract

Alcohol dehydrogenases belong to the large superfamily of medium-chain dehydrogenases/reductases, which occur throughout the biological world and are involved with many important metabolic routes. We considered the phylogeny of 190 ADH sequences of animals, fungi, and plants. Non-class III *Caenorhabditis elegans* ADHs were seen closely related to tetrameric fungal ADHs. ADH3 forms a sister group to amphibian, reptilian, avian and mammalian non-class III ADHs. In fishes, two main forms are identified: ADH1 and ADH3, whereas in amphibians there is a new ADH form (ADH8). ADH2 is found in Mammalia and Aves, and they formed a monophyletic group. Additionally, mammalian ADH4 seems to result from an ADH1 duplication, while in Fungi, ADH formed clusters based on types and genera. The plant ADH isoforms constitute a basal clade in relation to ADHs from animals. We identified amino acid residues responsible for functional divergence between ADH types in fungi, mammals, and fishes. In mammals, these differences occur mainly between ADH1/ADH4 and ADH3/ADH5, whereas functional divergence occurred in fungi between ADH1/ADH5, ADH5/ADH4, and ADH5/ADH3. In fishes, the forms also seem to be functionally divergent. The ADH family expansion exemplifies a neofunctionalization process where reiterative duplication events are related to new activities.

## Introduction

The alcohol dehydrogenase (ADH, EC 1.1.1.1) enzyme belongs to the large superfamily of medium-chain dehydrogenases/reductases, which include different enzyme activities, such as alcohol, sorbitol, xylitol, threonine dehydrogenase and quinone reductase (Persson *et al.*, 1993). Its activity appears to be universal in all life forms, derived from enzymes of separate family assignments and, frequently, involves multiple occurrences in a complex fashion (Norin *et al.*, 1997).

ADH class III (ADH3), with little or almost no ethanol activity and similar to the glutathione-dependent formaldehyde dehydrogenase, seems to be an ancestral form. Moreover, it has been characterized in vertebrates (Jörnvall and Höög, 1995; Hjelmqvist *et al.*, 1995b), invertebrates (Kaiser *et al.*, 1993; Danielsson *et al.*, 1994), plants (Martínez *et al.*, 1996), fungi (Sasnaukas *et al.*, 1992; Fernández *et al.*, 1995), and prokaryotes (Gutheil *et al.*, 1992; Ras *et al.*, 1995). ADH3 acts as a glutathione-dependent dehydrogenase in the oxidative elimination of formaldehyde, but does not function in ethanol or retinol oxidation, a function that is realized by other ADH classes (Duester *et al.*, 1999). Additionally, it is considered to be the most ancient form of vertebrate ADH, reflecting the fact that it is the only form also detected in invertebrates (Kaiser *et al.*, 1993).

Vertebrate ADH is a cytosolic, dimeric, zinc-containing, NAD-dependent enzyme with a subunit molecular mass of 40 kDa. Based on sequence alignment, phylogenetic analysis, catalytic properties and gene expression patterns at least eight distinct classes have been identified in vertebrates. ADH classes share around 60% amino acid sequence identity, and multiple ADH isoenzymes within a single class share above 90% identity (Jörnvall, 2008). They metabolize a wide variety of substrates, including ethanol, retinol, other aliphatic alcohols, hydroxysteroids, and lipid peroxidation products (Duester *et al.*, 1999).

In humans, ADH classes I (with three isoforms: A, B, and C, earlier called α, β, and γ, respectively), II, III, IV and V have been identified, and in mouse, classes I, II, III and IV have been described (Boleda *et al.*, 1993; Zheng *et al.*, 1993; Höög and Brandt 1995; Höög *et al.*, 2001). ADH class VI has been observed in rat and deer mouse (Zgombic-Knight *et al.*, 1995), and ADH class VII has been found in chicken (Kedishvili *et al.*, 1997), where it may act as a steroid/retinoid dehydrogenase. An amphibian ADH class VIII (class IV-like) has specificity towards NADP(H), with high catalytic efficiency specificity for retinoids and a high K_m_ for ethanol (Rosell *et al.*, 2003).

Several fungal and bacterial ADH enzymes are tetramers with two zinc atoms per monomer, while the animal and plant ADHs characterized to date are thought to be dimers also with two zinc atoms (Persson *et al.*, 2008). Five distinct ADHs are found in *Saccharomyces cerevisiae* and *Kluyveromyces*. ADH classes I and II of *S. cerevisiae* are cytoplasmic enzymes expressed under fermentative and respiratory conditions. Class III corresponds to a mitochondrial protein. Class IV is distantly related to the other four ADHs and is probably originated from a bacterium (Williamson and Paquin, 1987). Finally, class V was discovered during sequencing of the *S. cerevisiae* genome. The function of fungi classes III, IV and V is not completely understood (Wills and Jörnvall, 1979; Young *et al.*, 2000; Ladrière *et al.*, 2000; Thomson *et al.*, 2005).

In plants, the ADH gene family has been intensively studied in order to understand its genetics and molecular evolution. Generally, this family is characterized by a small number of copies and very diverse expression patterns. ADHs are involved in the energy production pathway, converting acetaldehyde into ethanol via fermentation during episodes of low oxygen concentrations or low temperatures (Dolferus *et al.*, 1994). Despite a large number of studies, there does not exist a clear correlation among ADH molecular evolution, function, and structure. Thompson *et al.* (2007) proposed that functional diversification during evolution has been responsible for site-specific shifts after ADH gene duplication in plants, and they furnished the first three-dimensional model of a plant ADH. Subsequently, they evaluated the impact of functional divergence on Poaceae, Brassicaceae, Fabaceae, and Pinaceae enzymes (Thompson *et al.*, 2010) and identified divergent amino acid residues in three important regions of plant ADH (the loop around the zinc ion, the region of monomer interactions and the active site).

In the present work we investigated the relationship among the different ADH classes of animals, fungi, and plants. Moreover, we identified the amino acid residues crucial for different types of functional divergence between duplicate genes using evolutionary and modeling tools in order to better understand the ADH diversification process.

## Materials and Methods

### Source of the data and sequence alignment

We obtained our protein data set from National Center of Biotechnology Information (NCBI). It consists of ADH amino acid sequences from the phyla Chordata (Classes Myxini, Actinopterygii, Elasmobranchii, Sarcopterygii, Amphibia, Reptilia, Aves, and Mammalia), Mollusca (Class Cephalopoda), Nematoda (Class Chromadorea), Platyhelminthes (Class Turbellaria), and Ascomycota (Classes Saccharomycetes, Sordariomycetes, and Eurotiomycetes). Plant amino acid sequences used in our previous studies (Thompson *et al.*, 2007) were incorporated in the analysis. Thus, 190 protein sequences composed the complete protein dataset. Moreover, we also downloaded 46 nucleotide alcohol dehydrogenase sequences from the NCBI server to evaluate the occurrence of positive selection. Protein alignments were performed using the PRANK software (Whelan and Goldman, 2001; Löytynoja and Goldman, 2005) with default settings. After manual inspection using Aliview (Larsson, 2014) software, we excluded the positions 40-74, 76-97 and 521-572. Furthermore, we used the TranslatorX (Abascal *et al.*, 2010) program to align DNA sequences based on their corresponding manually adjusted protein alignment. Alignments are available upon request.

### Phylogenetic analysis

We performed the selection of the best-fit models of amino acid for the maximum likelihood (ML) and Bayesian Inference (BI) analyses with the ProtTest program version 3.4.2 (Darriba *et al.*, 2011) using a fast strategy (optimization of model, branches, and topology of the tree) and without restricting the set of protein evolution candidate models. The program calculates a BIONJ tree, which is a distance based on a phylogeny reconstruction algorithm with better topological accuracy than Neighbor Joining (NJ) in all evolutionary conditions (Gascuel, 1997). The ProtTest program also uses the following criteria: Akaike Information Criterion (AIC, Akaike, 1974; Posada and Crandall, 2001), Corrected Akaike Information Criterion (AICc, Burnham and Anderson, 2003), Bayesian Information Criterion (BIC, Schwarz, 1978), and Decision Theory (DT). These criteria evaluate the relative importance and the model-averaged estimate of parameters. AICc and BIC include penalties for sample size. The jModelTest software (Posada, 2008) was used to evaluate the best evolutionary model for DNA sequences jointly with the use of the AIC, AICc, BIC, and DT criteria for the selection of the best model.

ADH phylogenies were estimated using Neighbor Joining (NJ; Saitou and Nei, 1987), available in the MEGA program version 6 (Kumar *et al.*, 2008, 2016), ML methods through the PhyML program (Phylogenetic Maximum Likelihood; Guindon and Gascuel, 2003), and BI using MrBayes version 3.2.4 (Ronquist *et al.*, 2012).

We applied p-distance, the Poisson-corrected amino acid distances, and the complete and pairwise deletion of gaps/missing data with 2,000 bootstrap repetitions to analyze the amino acid sequences using the NJ method. PhyML performed the analyses using the best models of protein and nucleotide sequence evolution that resulted from the ProtTest and jModelTest, respectively. This calculates an initial BIONJ tree and applies an approximate likelihood-ratio test (aLRT) for branch support. This approach is based on the conventional LRT principle. However, it is a faster test since the log-likelihood value *l*_*2*_ is computed by optimizing over the branch of interest and the four adjacent branches, whereas other parameters are fixed at their optimal values corresponding to the best ML tree (Anisimova and Gascuel, 2006). We used four chains of 1,000,000 generations, a burn-in of 25% as criteria, and the best evolutionary models identified by ProtTest and jModelTest for Bayesian inference. An average standard deviation of split frequencies equal to or smaller than 0.01 was the convergence criterion. The consensus tree was constructed considering a 50% majority rule consensus. Finally, we used FigTree version 1.4.2 and MEGA to visualize and edit the resulting phylogenies.

### Selection and functional diversification analysis

Branch lengths of the tree topologies were calculated using the M0 model available in the CODEML program of the PAML package (Yang, 2007) and, subsequently, the presence of positive selection was evaluated through the maximum likelihood models recommended by Yang (2007) using alcohol dehydrogenase DNA sequences. We carried out a series of LRTs to investigate whether ω was significantly different from 1 for each pairwise comparison: M1a *vs*. M2a, M0 *vs*. M3, and M7 *vs*. M8. LRT performs the comparison both with the constraint of ω=1 and without such constraint: LR=2(ln_1_-ln_2_). These LRT statistics approximately follow a chi-square distribution and the number of degrees of freedom is equal to the number of additional parameters in the more complex model (Anisimova *et al.*, 2001, 2002). We applied the Naive Empirical Bayes (NEB) and Bayes Empirical Bayes (BEB) approaches available in the PAML package to calculate the posterior probability that each site belongs to the positively selected class.

It is important to note that a relationship between a statistically detectable positive selection (ω>*1*) and functional divergence might not necessarily exist (Tennessen, 2008). Thus, to investigate further if any amino acid replacement could have led to adaptive functional diversification, we estimated the Type-I divergence by posterior analysis using DIVERGE version 3 (Gu and Vander Velden, 2002; Gu, 2006). The latter evaluates shifted evolutionary rates and altered amino acid properties after gene duplication (Gu, 2006). Type-I functional divergence (site-specific rate shift) refers to the evolutionary process resulting in site-specific rate shifts after gene duplication. It identifies amino acid residues highly conserved in one gene copy and highly variable in the other. The probability of a residue being under Type-I divergence is denoted θ_I_. *Q*_*I*_ *(k)* is the site *(k)*-specific score corresponding to the posterior probability that site *k* is related to type-I functional divergence (Zheng *et al.*, 2007).

Three-dimensional structures of alcohol dehydrogenase were downloaded from the RCSB Protein Data Bank (Berman *et al.*, 2000) to evaluate the impact of potential divergent amino acid residues. Moreover, PyMOL software version 1.8.4.2 was used to display and visualize *Homo sapiens* (ADH1, PDB ID 1HDX), *Saccharomyces cerevisiae* (ADH1, PDB ID 4W6Z), and *Gadus morhua* (ADH1, PDB ID 1CDO) structures.

## Results

### Phylogenetic analysis

In total, we performed a comparative phylogenetic analysis using 190 ADH amino acid sequences from animals, fungi and plants. The taxonomic classification, ADH types, accession numbers, and sequence sizes are shown in Table S1 (Supplementary Material). The best protein evolutionary model was LG (Le and Gascuel, 2008), with a proportion of invariable sites (+I) and rate variation among sites with a number of rate categories in the gamma distribution (+G), whereas GTR (Lanave *et al.*, 1984; Tavaré, 1986) with a gamma distribution (+G) was the best evolutionary model for DNA sequences.

**Figure 1:**
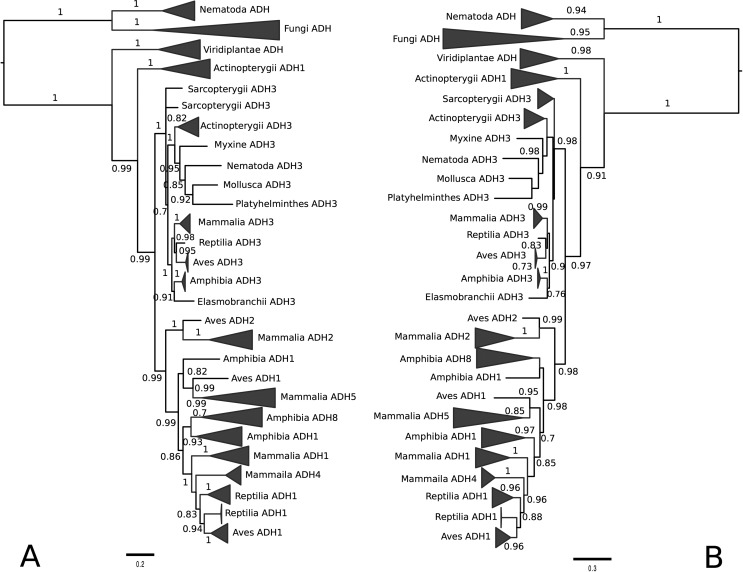
Evolutionary history of alcohol dehydrogenase proteins from plants, fungi and animals. A. Bayesian Inference; B. Maximum Likelihood. Numbers represent posterior probability and aLRT non-parametric branch support, respectively. Only values higher than 0.7 are shown. Scale bar indicates levels of sequence divergence.

The tree topologies resulting from the BI (Figure 1A) and ML (Figure 1B) methods do not differ significantly, especially when major clades are considered. We identified three monophyletic groups, corresponding to fungi, plants, and a larger group formed by animals. Additionally, we identified a clade composed by ADH sequences from the phylum Nematoda, which includes two *Caenorhabditis elegans* sequences (ADH1 and ADH2) that are placed close to the tetrameric fungal ADHs (Figure 1). Within a large group of ADH3s from animals it is interesting to note that *C. elegans* ADH3 clustered with those of *Octopus vulgaris* (Phylum Mollusca) and *Schmidtea mediterranea* (a freshwater planarian from Phylum Platyhelminthes). The invertebrate ADH3s formed a highly supported monophyletic group in BI phylogeny (Figure 1A). Mammalian, avian, reptilian, amphibian and Elasmobranch ADH3s also formed a monophyletic cluster (Figure 1).

**Figure 2:**
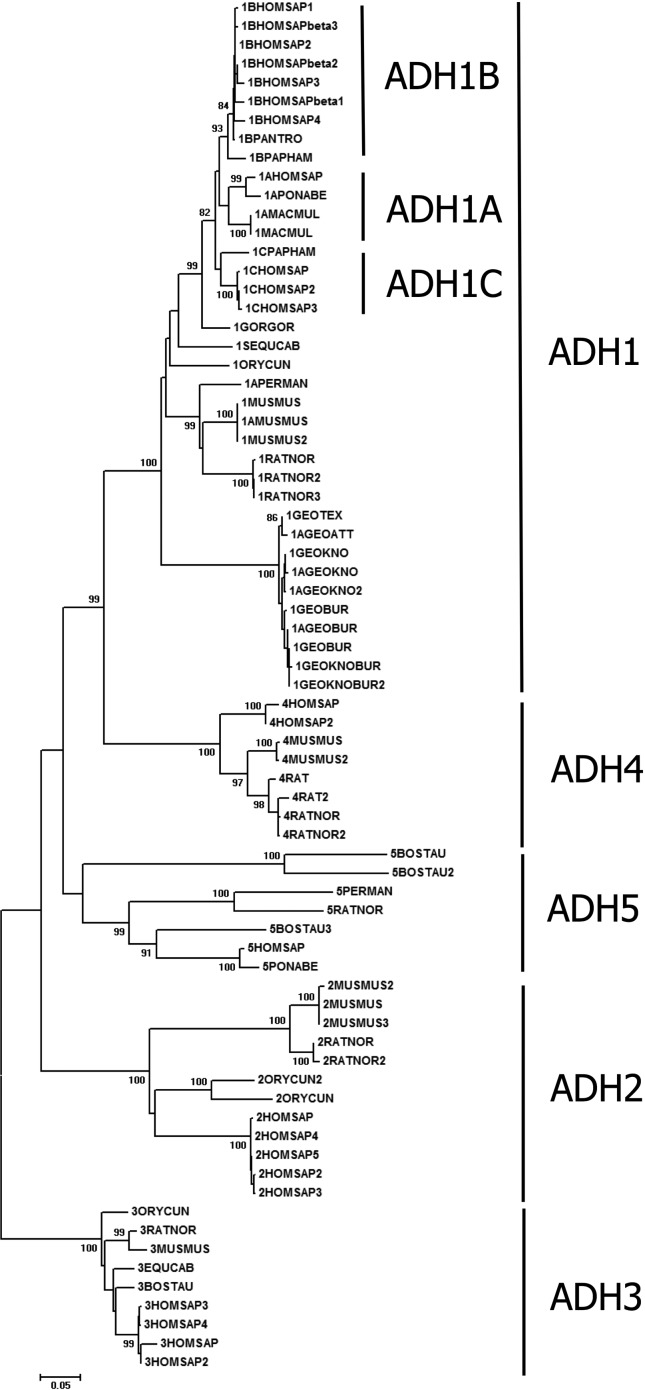
Phylogenetic tree of alcohol dehydrogenase proteins from mammals obtained by the neighbor-joining algorithm. Numbers represent bootstrap values; values higher than 80% are shown. Scale bar indicates levels of sequence divergence. Clusters distinguishable by ADH type are highlighted.

Most of the ADH1s were located in a large set that includes chordate ADH1, amphibian ADH8 and mammalian ADH4 and ADH5, with high bootstrap support for the individual clusters within the considered group (Figure 1). This form is the classical and highly variable liver enzyme responsible for ethanol metabolism. In fishes, we detected only two ADH groups: ADH3 and a second mixed class (here named ADH1, but also called ADH8 in the literature) that is separated from all other ADH1 forms (Figure 1 and Figure S1). Actinopterygian ADH1 seems to be basal to the highly supported clade formed by class III and non-class III ADHs (Figure 1). Mammalian ADH4s are highly similar to ADH1 in terms of primary sequence and are placed close to them in the phylogenetic tree (Figures 1 and 2). ADH2 is found in mammalian and avian/reptilian lineages, forming a sister group to tetrapod non-class III proteins (Figure 1). There was a distinct cluster of amphibian ADH8 close to Amphibian ADH1 (Figure 1 and Supplementary Material Figure S2) in the phylogenetic tree.

Phylogenetic relationships among mammalian ADH sequences are displayed in more detail in Figure 2, where monophyletic groups were formed according to ADH type. ADH1 showed sub-clusters (ADH1A, ADH1B, ADH1C), corresponding to different isoenzymes. Both in Figures 1 and 2, ADH4 was placed close to ADH1, suggesting that it originated from an ADH1 duplication. Mammalian ADH5 was placed close to avian ADH1 and, together with amphibian ADH1, formed a sister group in relation to a cluster that includes amphibian ADH1 and ADH8, mammalian ADH1 and ADH4, and ADH1 from Aves and Reptilia (Figure 1).

A new form (ADH8) appeared in amphibians, and it formed a separated cluster from ADH1 and ADH3 (Figure S2). Reptile ADH3 sequences formed a distinguishable group from ADH1 (Figure 1 and Figure S3). In addition to ADH1 and ADH3, ADH2 appears in the mammalian (Figure 2) and avian (Figure S4) lineages. ADH2 appears basal in relation to ADH1 in both mammals and birds (Figure 1), and ADH3 was basal to all sequences in these two animal groups.

**Figure 3:**
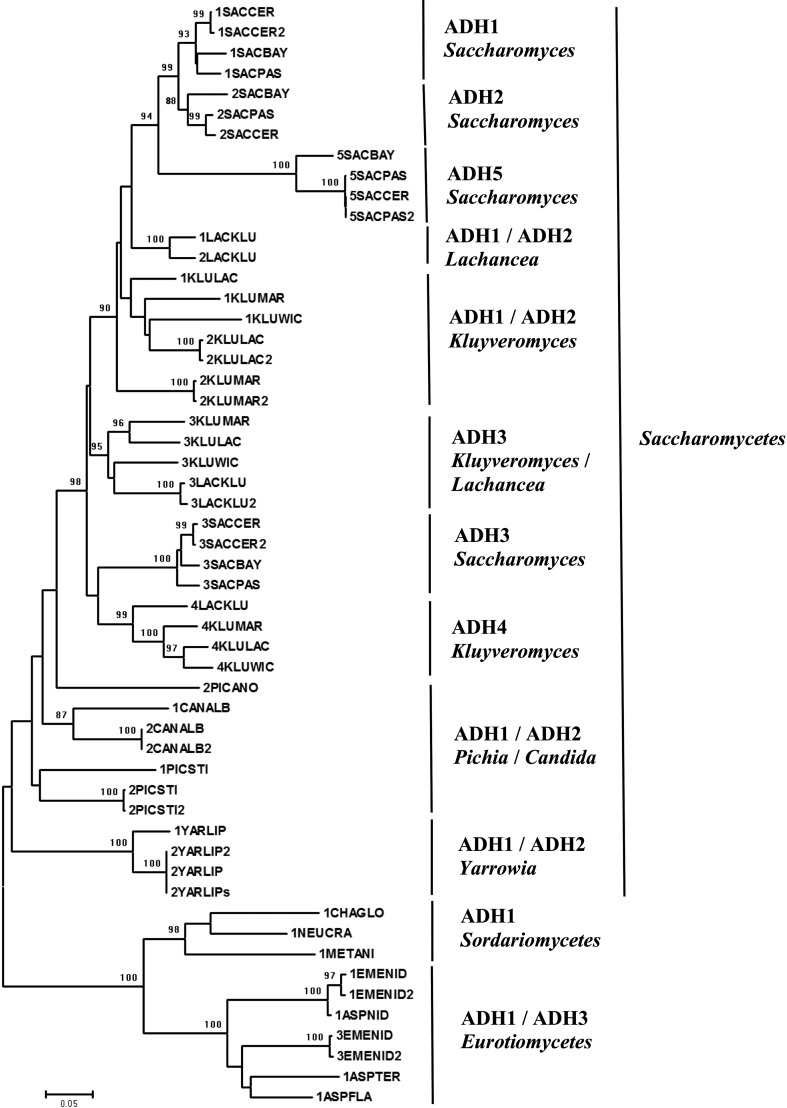
Phylogenetic tree of alcohol dehydrogenase proteins from fungi obtained by the neighbor-joining algorithm. Labels are indicating clusters distinguishable by ADH type and fungi genera. Numbers represent bootstrap values; values higher than 80% are shown. Scale bar indicates levels of sequence divergence.

A more complex pattern of sequence duplication was seen in fungi (Figure 3), where the ADH sequences clustered according to ADH type and fungi genera. A larger cluster composed by Saccharomycetes sequences is distinguishable. Additionally, Sordariomycetes and Eurotiomycetes ADHs formed distinct monophyletic groups. Our phylogenetic analysis grouped sequences from *Saccharomyces* by ADH type, with ADH2 closer to ADH1. *Saccharomyces* ADH1, ADH2 and ADH5 are probably derived from a common ancestor. ADH1 and ADH2 forms from *Lachancea* grouped together; ADH4 from *Kluyveromyces* formed a different group, as well as *Saccharomyces* ADH3 and ADH5. The *Yarrowia* and *Candida* sequences were also separated according to ADH type. *Pichia* ADHs did not form a monophyletic cluster, whereas ADH3 from *Kluyveromyces* and *Lachancea* clustered together.

### Selection and functional diversification analyses

Generally there was no indication of positive selection acting on *Adh* genes (Table 1), because LRTs comparing M1 (neutral) and M2 (selection), as well as M7 (beta) and M8 (beta & ω) were not statistically significant considering 0.01 as a cutoff. Additionally, the NEB and BEB approaches did not identify any site with posterior probability equal or higher than 0.95. However, the LRT comparing M0 (one-ratio) against M3 (discrete) was highly significant, indicating that selective pressure is highly variable among sites.

**Table 1:** Parameter estimates, likelihood scores under models of variable ω ratios among sites for alcohol dehydrogenase proteins.

Models^a^	lnL	2ΔL (df)	d_N_/d_S_ ^b^	Parameter estimates ^c^
M0: one-ratio (1)	-26849.05		0.1793	ω=0.1792
M3: discrete (5)	-26283.88	1130.34^*^ (4)	0.2097	*p*_0_=0.1556,*p*_1_=0.5393, (*p*_2_=0.3051) ω_0_=0.0137, ω_1_=0.1312, ω_2_=0.4485
M1a: nearly neutral (1)	-26577.11		0.3020	*p*_0_=0.8248, (*p*_1_=0.1752) ω_0_=0.1538, (ω1=1.0000)
M2a: positive selection (3)	-26577.11	0 (2)	0.3020	*p*_0_=0.8248,*p*_1_=0.1082, (*p*_2_=0.0670) ω_0_=0.1538, ω_1_=1.0000, ω_2_=1.0000
M7: β (2)	-26263.69		0.2186	*p*=0.8347,*q*=2.9362
M8: β & ω > 1 (4)	-26259.58	8.22 (2)	0.2261	*p*_0_=0.9548, (*p*_1_=0.0451) *p*=0.9630,*q*=4.044, ω=1.0000

a The number after the model code, in parentheses, is the number of free parameters in the ω distribution.

b This d_N_/d_S_ ratio is an average over all sites in the alcohol dehydrogenase gene alignment.

c Parameters in parentheses are not free parameters.

* Difference statistically significant when compared to the chi-squared distribution.

Coefficients of functional divergence (θ) of pairwise comparisons between mammalian, fishes, and fungal alcohol dehydrogenases are reported in Table 2. They showed statistically significant site-specific shifts of evolutionary rates, with θ varying markedly from 0.35 to 0.85. We used a site-specific profile based on the posterior probability (*Q*_*k*_) to identify amino acid residues responsible for functional divergences after gene duplication or speciation. To reduce false positives, a conservative cut-off value was empirically used: *Q*_*k*_ ≥ 0.90. Functionally important amino acid residue positions between the mammalian ADH forms and their respective *Q*_*k*_ values are shown in Table 3, whereas those important for the differentiation between fungi and fish forms are listed in Tables 4 and 5, respectively.

**Table 2:** Coefficients of functional divergence (θ) of pairwise comparisons in the alcohol dehydrogenase gene family.

Comparison	Group 1	Group 2	θ ± SE^a^	LRT^b^
Between forms	Mammals ADH3	Mammals ADH2	0.61 ± 0.21	7.90
	Mammals ADH3	Mammals ADH5	0.68 ± 0.19	12.98
	Mammals ADH2	Mammals ADH5	0.38 ± 0.15	6.57
	Mammals ADH2	Mammals ADH1	0.41 ± 0.11	14.10
	Mammals ADH5	Mammals ADH4	0.220.25	0.77*
	Mammals ADH5	Mammals ADH1	0.35 ± 0.11	9.74
	Mammals ADH4	Mammals ADH1	0.85 ± 0.19	19.18
	Fishes ADH1	Fishes ADH3	0.47 ± 0.08	30.47
	Fungi ADH1^S^	Fungi ADH3^S^	0.65 ± 0.26	6.11
	Fungi ADH1^S^	Fungi ADH5^S^	0.85 ± 0.12	50.71
	Fungi ADH3^S^	Fungi ADH5^S^	0.75 ± 0.15	24.85
	Fungi ADH1^S^	Fungi ADH4^K^	0.56 ± 0.18	9.46
	Fungi ADH1^S^	Fungi ADH3^KL^	0.46 ± 0.23	3.94
	Fungi ADH3^S^	Fungi ADH4^K^	0.07 ± 0.33	0.05*
	Fungi ADH3^S^	Fungi ADH3^KL^	0.001 ± 0.02	0*
	Fungi ADH5^S^	Fungi ADH4^K^	0.70 ± 0.10	47.53
	Fungi ADH5^S^	Fungi ADH3^KL^	0.74 ± 0.10	55.55
	Fungi ADH4^K^	Fungi ADH3^KL^	0.19 ± 0.15	1.58*

a SE stands for standard error.

b LRT: Likelihood Ratio Test. All values are statistically significant at *P* < 0.05 or less, when compared to the chi-squared distribution with one degree of freedom, except those labeled with (*). Sequences of birds, amphibians and reptilians had incomplete information for this type of analysis.

**Table 3:** Amino acid residues important for the functional divergence between mammalian ADH forms.

Amino acid residues^a^	ADH1/ADH4	ADH1/ADH2	ADH5/ADH3	ADH3/ADH2
44 (Val41)			0.91	
54 (His51)		0.92		
63	0.92			
64	0.91			
68	0.93			
77	0.92			
84	0.93			
99	0.94			
102	0.92			
109	0.92			
112	0.93			
**122 (Leu112)**	**0.95**			
123	0.92			
124	0.90			
138	0.90			
142	0.91			
147	0.93			
152	0.93			
155	0.92			
157	0.92			
163	0.92			
166	0.93			
171	0.92			
174	0.92			
183	0.93			
205	0.91			
220	0.92			
228 (Ala213)			0.91	
239	0.93			
246 (Lys231)			0.90	
248	0.93			
**253 (Thr238)**			**0.96**	0.93
257	0.93			
261	0.93			
262	0.91			
271	0.93			
280	0.92			
281	0.93			

a In bold are amino acid residues with *Q*(*k*) ≥ 0.95. The correspondent amino acid residues in the three-dimensional structure of human ADH1 (PDB ID 1HDX, Figure 4A) are indicated.

**Table 4:** Amino acid residues important for the functional divergence between fungal ADH forms.

Amino acid residues^a^	ADH1^S^/ADH5^S^	ADH5^S^/ADH4^K^	ADH5^S^/ADH3^KL^
49	**0.95**	0.87	
60	0.94		**0.95**
69	0.94		**0.95**
70	0.94	0.91	**0.95**
73	**0.96**		
76	0.94		**0.95**
97	0.94	0.91	**0.95**
104	**0.95**	0.87	
120	**0.95**		
126 (Lys80)		**0.96**	
131	**0.95**		0.93
181	**0.96**		
187		0.91	**0.95**
188	**0.95**		
192	0.94	0.91	**0.95**
195	**0.95**		
196	**0.96**	0.87	
199	**0.95**		
204	0.94	0.91	**0.95**
215	0.94	0.91	**0.95**
216	**0.96**	**0.97**	
219	**0.96**		
239	0.94	0.91	**0.95**
246	**0.99**		
255	0.94	0.91	**0.95**
267	**0.95**		
271 (Lys223)		0.91	**0.95**
272 (Glu224)		0.87	**0.96**
279 (Gly229)		0.87	**0.95**
280 (Ala230)		0.87	**0.96**
282	**0.96**		
298	**0.96**		
315	**0.96**		
320 (Thr264)		**0.97**	
329	**0.95**		
333	0.94	0.91	**0.95**

a In bold are amino acid residues with *Q*(*k*) ≥ 0.95.

S *Saccharomyces*;

K *Kluyveromyces* ADH4;

KL *Kluyveromyces* / *Lachancea*. The correspondent amino acid residues in the three-dimensional structure of yeast ADH1 (PDB ID 4W6Z, Figure 4B) are indicated.

**Table 5:** Amino acid residues important for the functional divergence between ADH forms of fishes.

Amino acid residues^a^	ADH1/ADH3
130 (Glu128)	0.88
234 (Lys232)	0.88
302 (Leu298)	**0.91**
328 (Gly324)	**0.93**
355 (Pro351)	**0.93**

a In bold are amino acid residues with *Q*(*k*) ≥ 0.90. The correspondent amino acid residues in the three-dimensional structure of cod ADH1 (PDB ID 1CDO, Figure 4C) are indicated.

For mammals (Table 3), one site (253) seemed to be especially important for the differences between ADH3/ADH2 and ADH3/ADH5. The sites 44, 228 and 246 were also identified as divergent for ADH5/ADH3, whereas site number 54 was so for ADH1/ADH2. A number of differences in functionally important sites occurred mainly between ADH4 and ADH1 (33 sites) and ADH3/ADH5 (4 sites). Site number 122 showed a *Q*_*k*_ =0.95 for the ADH1/ADH4 comparison. We located sites 44, 54, 122, 228, 246 and 253 in the three-dimensional structure of human ADH1 (PDB ID: 1HDX, corresponding to sequence AAA51884; Table S1). They were located in a β-strand, an α-helix near NAD, a coil close to a zinc ion, and in a coil, α-helix, and β-strand in the molecular surface, respectively (Figure 4A).

**Figure 4:**
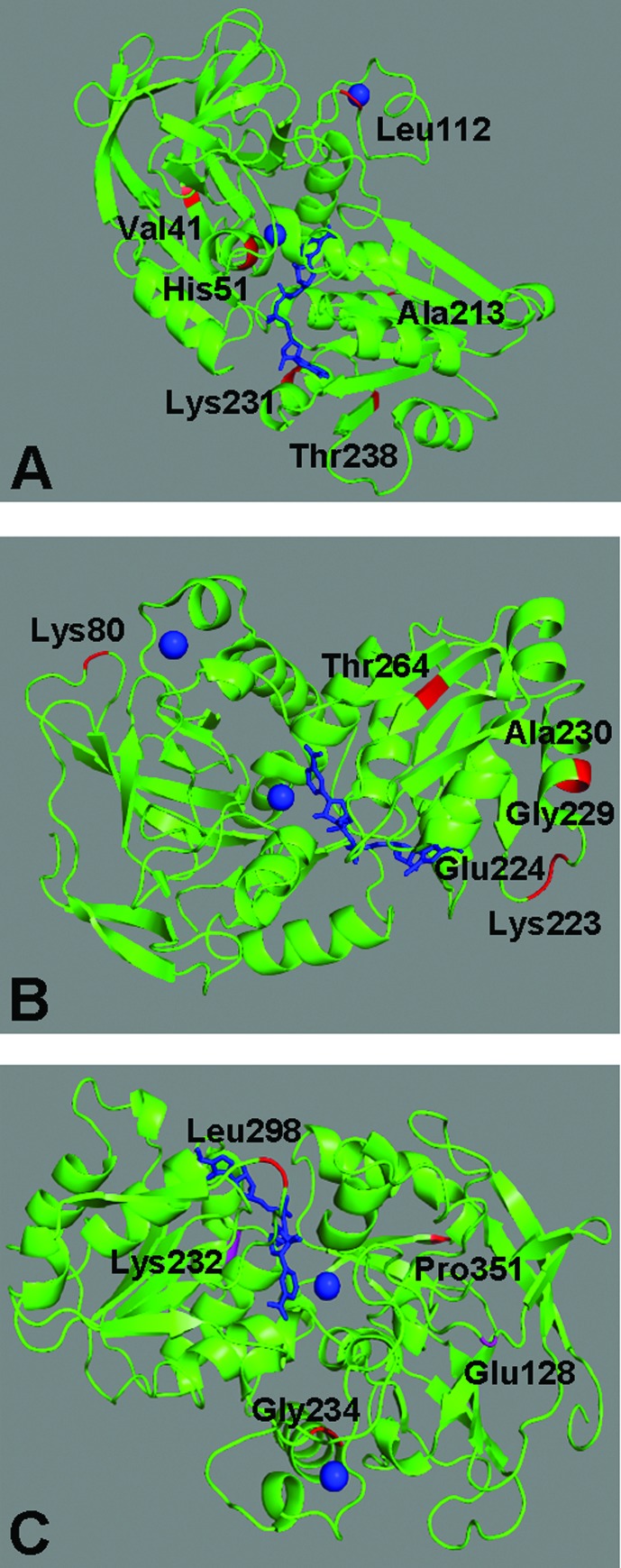
Three-dimensional structures of alcohol dehydrogenase from: A. *Homo sapiens* (ADH1, PDB ID 1HDX, chain A), B. *Saccharomyces cerevisiae* (ADH1, PDB ID 4W6Z, chain A), and C. *Gadus morhua* (ADH1, PDB ID 1CDO chain A). Blue spheres = zinc ions. Blue bars = nicotinamide-adenine dinucleotide (NAD) in A and C, and nicotinamide-8-iodo-adenine dinucleotide (8ID) in B. Amino acids responsible for functional divergence and their respective position in the PDBs are indicated in the figures.

For fungi (Table 4), several sites accounted for differences between ADH1 and ADH5 from *Saccharomyces*. In fact, there are 18 sites, considering a *Q*_*k*_ ≥ 0.95. Additionally, ADH5 from this fungus was identified as functional divergent from ADH4 from *Kluyveromyces* (ADH4^K^) and ADH3 from *Kluyveromyces* and *Lachancea* (ADH3^KL^). These sites, 271, 272, 279, and 280 (*Q*_*k*_ ≥ 0.95), were likely responsible for the divergence between ADH3^KL^ and ADH5^S^, whereas sites number 126 and 320 (*Q*_*k*_ ≥ 0.95) are associated with that between ADH4^K^/ADH5^S^. We identified these amino acids in the three-dimensional structure of *Saccharomyces cerevisiae* ADH1 (PDB ID 4W6Z, chain A). The sites 126, 271, 272, 279, and 280 corresponding to coil, coil, coil and an α-helix, respectively, are all located in the protein molecular surface, whereas site 320 corresponds to a β-strand important to interaction with chain B of this tetrameric ADH (Figure 4B).

ADH1 and ADH3 from fishes are also functionally divergent, as indicated by the *Q*_*k*_ values for specific amino acids. The sites 302, 328 and 355 all showed a *Q*_*k*_ ≥ 0.90 (Table 5). They were identified in the 3D structure of *Gadus morhua* ADH3 (PDB ID 1CDO, chain A). The first two are close to the NAD (nicotinamide-adenine-dinucleotide) coenzyme, while the site number 355 is in a coil in the molecular surface (Figure 4C).

## Discussion

Gene duplication is an important precursor of evolutionary diversification. The majority of new genes originate through duplication, chromosomal rearrangement, and the subsequent divergence of pre-existing genes (Lawton-Rauth, 2003). The existence of several multigenic families is an indication of the importance of gene duplication in the origin of function novelties (Wendel, 2000). Phylogenetic analysis has been a powerful approach to investigate the role of gene duplications in evolution.

The alcohol dehydrogenase enzymes form a large and diverse family that has contributed to the understanding of protein evolution, enzymatic mechanisms, metabolic functions, and regulatory roles. They show chemically modified sub-forms, isoenzymes, classes, and separate enzymes, presenting a wide range of distinct functions, as well as redundancy with overlaps in activity (Jörnvall, 2008). We have theoretically demonstrated that different plant ADH forms may be submitted to an evolutionary diversification process that occurred after gene duplication (Thompson *et al.*, 2007, 2010). The next step was to evaluate the importance of this process in ADHs of other organisms, to obtain a comprehensive panorama for ADH molecular evolution.

We identified three monophyletic groups composed by fungi, plants and animals. Glasner *et al.* (1995) analyzed a smaller number of sequences (22) and found a similar pattern of evolution for these proteins. The identification of two *Caenorhabditis elegans* sequences (ADH1 and ADH2) close to the tetrameric fungal ADHs (Figure 1) agrees with that obtained by Glasner *et al.* (1995) who described for the first time fungal-like ADH sequences among metazoans. Both *C. elegans* ADH forms show ethanol degradation activity, preferentially for longer alcohols. It may be possible that additional fungal-like sequences will be discovered in other animals or plants, which could be explained by one or multiple deletions in lineages generating the modern plants and animals, or it may be the result of convergent evolution (Glasner *et al.*, 1995).

We also identified a close evolutionary relationship among ADH3s from *C. elegans*, *Octopus vulgaris,* and *Schmidtea mediterranea*, a freshwater planarian, within the large group of ADH3s from all animals. Godoy *et al.* (2007) also found a close relationship between the *S. mediterranea* and *C. elegans* ADH3s. Kaiser *et al.* (1993) described the *O. vulgaris* ADH3, which was the first-detected group of animals that lack ethanol dehydrogenase activity. No other ADH classes are present in planarians also, as suggested by *in silico* analysis that indicated that only one contig was sufficient to account for the cDNA and 40 trace sequences from the planarian databases (Godoy *et al.*, 2007).

We observed a monophyletic cluster of ADH3 (Figure 1) in this work. This enzyme is widely known as a glutathione-dependent formaldehyde dehydrogenase that can oxidize ethanol at high concentrations (Dasmahapatra *et al.*, 2001) but preferentially metabolizes longer aliphatic and aromatic alcohols (Reimers *et al.*, 2004). ADH3 has been described as a ubiquitous enzyme in vertebrates (Funkenstein and Jakowlew, 1996), with a spatio-temporal regulation in zebrafish development (Dasmahapatra *et al.*, 2001; Cañestro *et al.*, 2003). Additionally, ADH3 is found in the cell nucleus, where it may have a probable DNA protection function (Iborra *et al.*, 1992; Fernández *et al.*, 2003), differently from the other ADHs, which commonly have a cytosolic location (Gonzàlez-Duarte and Albalat, 2005). In invertebrates, its expression is mainly found in digestive tissues (Godoy *et al.*, 2007). We demonstrated that sites 44, 228, 246, and 253 seem to be fundamental for the divergence of ADH3/ADH5 and ADH3/ADH2 in mammals.

ADH1 is the classical liver enzyme responsible for ethanol metabolism. In fishes we identified ADH3 and a second mixed class (as previously remarked, it is here named ADH1, but also called ADH8 in the literature) that is structurally similar to class III but functionally similar to ADH1 (the classical alcohol-metabolizing enzyme; Dasmahapatra *et al.*, 2005). This hybrid characteristic may explain why the Actinopterygii ADH1 cluster is separated from all other ADH1s (Figure 1). Fishes constitute the first vertebrate class with documented expression of more than one ADH class (Dasmahapatra *et al.*, 2005). In this report we identified some amino acid residues important for functional differentiation between ADH1 and ADH3. They are located in regions of functional importance, such as those close to the NAD coenzyme and the zinc ion.

ADH1 has tissue-specific expression and is involved in different metabolic pathways, such as ethanol oxidation, norepinephrine, dopamine, serotonin and bile acid metabolism (Höög *et al.*, 2001), oxidation of retinol *in vitro* (Boleda *et al.*, 1993) and *in vivo* (Deltour *et al.*, 1999). It is highly expressed in the liver and also significantly expressed in the uterus, adrenal, small and large intestine, kidney, testis, and epididymis (Gonzàlez-Duarte and Albalat, 2005)**.** The ADH1 structure has three conserved positions, His67, Glu68, and Phe140, which have been proposed as a signature for class assignment (Norin *et al.*, 1997), and three variable segments near the substrate-binding pocket and the subunit interaction region. In contrast, these regions are among the most conserved in ADH3 (Cañestro *et al.*, 2003). It is important to note that preservation of those previously cited conserved amino acids does not necessarily imply ethanol-oxidizing activity (Reimers *et al.*, 2004). Additionally, there are two main domain conformations of ADH1 described as ‘open’ in the apoenzyme and ‘closed’ in the binary and ternary complexes. Different substrate specificity and kinetic mechanisms of ADH1 and ADH3 may be due to these ‘open’ and ‘closed’ conformations (Sanghani *et al.*, 2002).

Mammalian ADH4s were placed close to ADH1 in the phylogenetic tree (Figures 1 and 2), which suggests that it originated from ADH1 duplication. Our results corroborated the hypothesis proposed by Gonzàlez-Duarte and Albalat (2005) that ADH4 may be the result of a mammalian-specific *Adh1* duplication, since this class has not been detected in birds or reptilians (Figure 1). Estonius *et al.* (1994), Parés *et al.* (1994) and Strömberg and Höög (2000) obtained similar results. In mammals, ADH4 is specifically expressed in epithelial tissues, such as stomach mucosa (Parés *et al.*, 1994). ADH4 functions in retinoid oxidation *in vitro* (Boleda *et al.*, 1993). However, ADH4-null mutant mice showed weak phenotypic effects, which may indicate a contribution in specific routes, not involved in systemic retinol metabolism (Deltour *et al.*, 1999). In this work we identify a significant functional divergence of mammalian ADH4 and ADH1, with some amino acid residues of these differences located in functional important regions, such as site no. 122 close to the zinc ion.

ADH2 was found in mammalian and avian/reptilian lineages forming a sister group to tetrapod non-class III proteins, reinforcing the results of Hjelmqvist *et al.* (1995b). Based on the phylogenetic analysis, as well as biochemical and structural characteristics (Höög *et al.*, 2001, Gonzàlez-Duarte and Albalat, 2005), it is reasonable to suggest that ADH2 is derived from a tetrapod ADH3. ADH2 proteins have higher K_m_ values toward ethanol and preferentially metabolize larger aliphatic and aromatic alcohols/aldehydes (Reimers *et al.*, 2004). Moreover, they are structurally more divergent than the ADH1 forms, for which variation is classically known (Hjelmqvist *et al.*, 1995a). A functionally important site (54, close to the zinc ion in the ADH three-dimensional structure) seems to be important for ADH1/ADH2 divergence in mammals.

Amphibian ADH8 formed a distinct cluster, which confirms the distinct characteristics of ADH8, such as a large active site pocket, very different proton-relay pathway, very specific rearrangements in the phosphate-binding site cofactor, and weak interactions of the adenine moiety (Rosell *et al.*, 2003). This form has a unique NADP(H) specificity and was first described as ADH4-like. However, these characteristics led to its classification in a new class (Rosell *et al.*, 2003).

In relation to the alcohol dehydrogenases from fungi, the ADH1-ADH2 duplication seems to have occurred before the divergence of the *Saccharomyces* species and after the divergence between *Saccharomyces* and *Kluyveromyces,* which has been estimated to have occurred 80 ± 15 million years ago (Thomson *et al.*, 2005). Indeed *Saccharomyces* ADH1, ADH2 and ADH5 probably derived from a common ancestor, as suggested by Ladrière *et al.* (2000). Moreover, ADH5 has the highest rate of ADH sequence divergence. In this report, ADH5 was shown to be functionally divergent from ADH1. *Saccharomyces* ADH1 and ADH2 are cytoplasmatic enzymes acting in the fermentation and gluconeogenesis processes, respectively, while ADH3 is located in the mitochondria (de Smidt *et al.*, 2008). *Kluyveromyces* ADH has two cytoplasmatic (ADH1 and ADH2) and two mitochondrial (ADH3 and ADH4) enzymes. In the present work, we have shown that ADH4^K^ and ADH3^KL^ are functionally divergent from *Saccharomyces* ADH5. We recall that Lertwattanasakul *et al.* (2007) have proposed that *Kluyveromyces marxianus* ADHs have distinct roles in cells, because the different *Adh* genes are differentially expressed depending on growth phase and carbon source. Since the *Saccharomyces* and *Kluyveromyces* genomes are similar, while their ADH sequences have been submitted to different rates of divergence (Ladrière *et al.*, 2000), they may have a lower structural constraint or submission to a functionally divergence process, and this could lead to new enzyme functions. Indeed, this seems to occur in animals (Höög *et al.*, 2001) and was theoretically demonstrated in plants (Thompson *et al.*, 2007).

Natural selection has been described as responsible for the evolution of many genes (Hey, 1999). A widely used method to detect positive selection is through the ratio of nonsynonymous to synonymous rates (ω *= d*_*N*_ */d*_*S*_). It is assumed that synonymous substitutions are neutral, whereas the nonsynonymous are subject to selection. Consequently, a ω statistically higher than 1 would indicate the action of positive selection or a relaxed selective constraint, whereas low *d*_*N*_ */d*_*S*_ values would mean conservation of the gene product due to purifying selection (Tennessen, 2008). Although we did not directly identify positive selection acting on the alcohol dehydrogenase genes, there appears to be variable selective pressure acting among sites, as indicated by LRT when the M0 (one-ratio) and M3 (discrete) models are compared. Therefore, we tested if any amino acid replacement could have led to adaptive functional diversification and the results indicated that there are some sites in different species that exhibit different evolutionary rates and altered amino acid properties after gene duplication, but experimental structural-functional studies are mainly restricted to the ADH1 and ADH3 enzymes. Future theoretical and experimental studies are needed to establish the impact of these amino acid replacements in the ADH structure and function. For instance, docking and molecular dynamics simulations could add valuable information about the functional divergence of these proteins.

## Supplementary material

The following online material is available for this article:

Table S1 - Alcohol dehydrogenase proteins used in this study.

Figure S1 - Evolutionary relationships of fish ADH proteins.

Figure S2 - Evolutionary relationships of amphibian ADH proteins.

Figure S3 - Evolutionary relationships of reptilian ADH proteins.

Figure S4 - Evolutionary relationships of avian ADH proteins.

## References

[B1] Abascal F, Zardoya R, Telford MJ (2010). TranslatorX: Multiple alignment of nucleotide sequences guided by
amino acid translations. Nucleic Acids Res.

[B2] Akaike H (1974). A new look at the statistical model
identification. IEEE Trans Autom Control.

[B3] Anisimova M, Gascuel O (2006). Approximate likelihood-ratio test for branches: A fast, accurate,
and powerful alternative. Syst Biol.

[B4] Anisimova M, Bielawski JP, Yang Z (2001). Accuracy and power of the likelihood ratio testing in detecting
adaptive molecular evolution. Mol Biol Evol.

[B5] Anisimova M, Bielawski JP, Yang Z (2002). Accuracy and power of Bayes prediction of amino acid sites under
positive selection. Mol Biol Evol.

[B6] Berman HM, Westbrook J, Feng Z, Gilliland G, Bhat TN, Weissig H, Shindyalov IN, Bourne PE (2000). The protein data bank. Nucleic Acids Res.

[B7] Boleda MD, Saubi N, Farrés J, Parés X (1993). Physiological substrates for rat alcohol dehydrogenase classes:
Aldehydes of lipid peroxidation, omega-hydroxyfatty acids, and
retinoids. Arch Biochem Biophys.

[B8] Burnham KP, Anderson DR (2003). Multimodel inference: Understanding AIC and BIC in model
selection. Sociol Method Res.

[B9] Cañestro C, Godoy L, Gonzàlez-Duarte R, Albalat R (2003). Comparative expression analysis of *Adh3* during
arthropod, urochordate, cephalochordate and vertebrate development
challenges its predicted housekeeping role. Evol Dev.

[B10] Danielsson O, Atrian S, Luque T, Hjelmqvist L, Gonzalez-Duarte R, Jörnvall H (1994). Fundamental molecular differences between alcohol dehydrogenase
classes. Proc Natl Acad Sci U S A.

[B11] Darriba D, Taboada GL, Doallo R, Posada D (2011). ProtTest 3: Fast selection of best-fit models of protein
evolution. Bioinformatics.

[B12] Dasmahapatra AK, Doucet HL, Bhattacharyya C, Carvan MJ (2001). Developmental expression of alcohol dehydrogenase (ADH3) in
zebrafish (*Danio rerio*). Biochem Biophys Res Commun.

[B13] Dasmahapatra AK, Wang X, Haasch ML (2005). Expression of *Adh8* mRNA is developmentally
regulated in Japanese medaka (*Oryzias
latipes*). Comp Biochem Physiol.

[B14] Deltour L, Foglio MH, Duester G (1999). Impaired retinol utilization in *Adh4* alcohol
dehydrogenase mutant mice. Dev Genet.

[B15] de Smidt O, du Preez JC, Albertyn J (2008). The alcohol dehydrogenases of *Saccharomyces
cerevisae:* a comprehensive review. FEMS Yeast Res.

[B16] Dolferus R, Jacobs M, Peacock WJ, Dennis ES (1994). Differential interactions of promoter elements in stress
responses of *Arabidopsis Adh* gene. Plant Physiol.

[B17] Duester G, Farrés J, Felder MR, Holmes RS, Höög JO, Parés X, Plapp BV, Yin SJ, Jörnvall H (1999). Recommended nomenclature for the vertebrate alcohol dehydrogenase
gene family. Biochem Pharmacol.

[B18] Estonius M, Hjelmqvist L, Jörnvall H (1994). Diversity of vertebrate class I alcohol dehydrogenase: mammalian
and non-mammalian enzyme functions correlated through the structure f a
ratite enzyme. Eur J Biochem.

[B19] Fernández MR, Biosca JA, Norin A, Jörnvall H, Parés X (1995). Class III alcohol dehydrogenase from *Saccharomyces
cerevisae*: Structural and enzymatic features differ toward the
human/mammalian forms in a manner consistent with functional needs in
formaldehyde detoxication. FEBS Lett.

[B20] Fernández MR, Biosca JA, Parés X (2003). S-nitrosoglutathione reductase activity of human and yeast
glutathione-dependent formaldehyde dehydrogenase and its nuclear and
cytoplasmic localization. Cell Mol Life Sci.

[B21] Funkenstein B, Jakowlew SB (1996). Molecular cloning of fish alcohol dehydrogenase
cDNA. Gene.

[B22] Gascuel O (1997). BIONJ: An improved version of the NJ algorithm based on a simple
model of sequence data. Mol Biol Evol.

[B23] Glasner JD, Kocher TD, Collins JJ (1995). *Caenorhabditis elegans* contains genes encoding two new
members of the Zn-containing alcohol dehydrogenase family. J Mol Evol.

[B24] Godoy L, Gonzàlez-Duarte R, Albalat R (2007). Analysis of planarian *Adh3* supports an
intron-rich architecture and tissue-specific expression for the urbilaterian
ancestral form. Comp Biochem Physiol.

[B25] Gonzàlez-Duarte R, Albalat R (2005). Merging protein, gene and genomic data: The evolution of the
MDR-ADH family. Heredity.

[B26] Gu X (2006). A simple statistical method for estimating type-II
(cluster-specific) functional divergence of protein
sequences. Mol Biol Evol.

[B27] Gu X, Vander Velden K (2002). DIVERGE: Phylogeny-based analysis for functional-structural
divergence of a protein family. Bioinformatics.

[B28] Guindon S, Gascuel O (2003). A simple, fast, and accurate algorithm to estimate large
phylogenies by maximum likelihood. Syst Biol.

[B29] Gutheil WG, Holmquist B, Vallee BL (1992). Purification, characterization, and partial sequence of the
glutathione-dependent formaldehyde dehydrogenase from *Escherichia
coli*: a class III alcohol dehydrogenase. Biochemistry.

[B30] Hey J (1999). The neutralist, the fly, and the selectionist. Trends Ecol Evol.

[B31] Hjelmqvist L, Estonius M, Jörnvall H (1995a). The vertebrate alcohol dehydrogenase system: Variable class II
type form elucidates separate stages of enzymogenesis. Proc Natl Acad Sci U S A.

[B32] Hjelmqvist L, Shafqat J, Siddiqi AR, Jörnvall H (1995b). Alcohol dehydrogenase of class III: Consistent patterns of
structural and functional conservation in relation to class I and other
proteins. FEBS Lett.

[B33] Höög JO, Brandt M (1995). Mammalian class VI alcohol dehydrogenase: Novel types of the
rodent enzymes. Adv Exp Med Biol.

[B34] Höög JO, Hedberg JJ, Stromberg P, Svesson S (2001). Mammalian alcohol dehydrogenase – functional and structural
implications. J Biomed Sci.

[B35] Iborra FJ, Renau-Piqueras J, Portoles M, Boleda MD, Guerri C, Parés X (1992). Immunocytochemical and biochemical demonstration of formaldehyde
dehydrogenase (class III alcohol dehydrogenase) in the
nucleus. J Histochem Cytochem.

[B36] Jörnvall H (2008). MDR and SDR gene and protein superfamilies. Cell Mol Life Sci.

[B37] Jörnvall H, Höög JO (1995). Nomenclature of alcohol dehydrogenases. Alcohol Alcoholism.

[B38] Kaiser R, Férnandez MR, Parés X, Jörnvall H (1993). Origin of human alcohol dehydrogenase system: Implications from
the structure and properties of the octopus protein. Proc Natl Acad Sci U S A.

[B39] Kedishvili NY, Gough WH, Chernoff EAG, Hurley TD, Stone CL, Bowman KD, Popov KM, Bosron WF, Li TK (1997). cDNA sequence and catalytic properties of a chick embryo alcohol
dehydrogenase that oxidizes retinol and
3b,5a-hydroxysteroids. J Biol Chem.

[B40] Kumar S, Dudley J, Nei M, Tamura K (2008). MEGA: A biologist centric software for evolutionary analysis of
DNA and protein sequences. Brief Bioninform.

[B41] Kumar S, Stecher G, Tamura K (2016). MEGA7: Molecular Evolutionary Genetics Analysis version 7.0 for
bigger datasets. Mol Biol Evol.

[B42] Ladrière JM, Georis I, Guérineau M, Vandenhaute J (2000). *Kluyveromyces marxianus* exhibits an ancestral
*Saccharomyces cerevisiae* genome organization downstream
of ADH2. Gene.

[B43] Lanave C, Preparata G, Sacone C, Serio G (1984). A new method for calculating evolutionary substitution
rates. J Mol Evol.

[B44] Larsson A (2014). AliView: A fast and lightweight alignment viewer and editor for
large data sets. Bioinformatics.

[B45] Lawton-Rauth A (2003). Evolutionary dynamics of duplicated genes in
plants. Mol Phylogenet Evol.

[B46] Le SQ, Gascuel O (2008). LG: An improved, general amino-acid replacement
matrix. Mol Biol Evol.

[B47] Lertwattanasakul N, Sootsuwan K, Limtong S, Thanonkeo P, Yamada M (2007). Comparison of the gene expression patterns of alcohol
dehydrogenase isozymes in the termotolerant yeast *Kluyveromyces
marxianus* and their physiological functions. Biosci Biotechnol Biochem.

[B48] Löytynoja A, Goldman N (2005). An algorithm for progressive multiple alignment of sequences with
insertions. Proc Natl Acad Sci U S A.

[B49] Martínez MC, Achkor H, Persson B, Férnandez MR, Shafqat J, Farrés J, Jörnvall H, Parés X (1996). *Arabidopsis* formaldehyde dehydrogenase. Eur J Biochem.

[B50] Norin A, Van Ophen PW, Piersma SR, Persson B, Duine JA, Jörnvall H (1997). Mycothiol-dependent formaldehyde dehydrogenase, a prokaryotic
medium-chain dehydrogenase/reductase, phylogenetically links different
eukaryotic alcohol dehydrogenases. Eur J Biochem.

[B51] Parés X, Cederlund E, Moreno A, Hjelmqvist L, Farrés J, Jörnvall H (1994). Mammalian class IV alcohol dehydrogenase (stomach alcohol
dehydrogenase): structure, origin, and correlation with
enzymology. Proc Natl Acad Sci U S A.

[B52] Persson B, Bergman T, Keung WM, Waldenström U, Holmquist B, Vallee BL, Jörnvall H (1993). Basic features of class-I alcohol dehydrogenase: variable and
constant segments coordinated by inter-class and intra-class
variability. Conclusions from characterization of the alligator enzyme. Eur J
Biochem.

[B53] Persson B, Hedlund J, Jörnvall H (2008). The MDR superfamily. Cell Mol Life Sci.

[B54] Posada D (2008). jModelTest: Phylogenetic model averaging. Mol Biol Evol.

[B55] Posada D, Crandall KA (2001). Selecting the best-fit model of nucleotide
substitution. Syst Biol.

[B56] Ras J, Van Ophem PW, Reijnders WNM, Van Spanning RJM, Duine JA, Stouthamer AH, Harms N (1995). Isolation, sequencing, and mutagenesis of the gene encoding NAD-
and glutathione-dependent formaldehyde dehydrogenase (GD-FALDH) from
*Paracoccus denitrificans*, in which GD-FALDH is
essential for methylotrophic growth. J Bacteriol.

[B57] Reimers MJ, Hahn ME, Tanguay RL (2004). Two zebrafish alcohol dehydrogenases share common ancestry with
mammalian class I, II, IV, and V alcohol dehydrogenase genes but have
distinct functional characteristics. J Biol Chem.

[B58] Ronquist F, Teslenko M, van der Mark P, Ayres D, Darling A, Höhna S, Larget B, Liu L, Suchard MA, Huelsenbeck JP (2012). MrBayes 3.2: Efficient Bayesian phylogenetic inference and model
choice across a large model space. Syst Biol.

[B59] Rosell A, Valencia E, Parés X, Fita I, Farrés J, Ochoa WF (2003). Crystal structure of the vertebrate NADP(H)-dependent alcohol
dehydrogenase (ADH8). J Mol Biol.

[B60] Saitou N, Nei M (1987). The neighbor-joining method: A new method for reconstructing
phylogenetic trees. Mol Biol Evol.

[B61] Sanghani PC, Bosron WF, Hurley TD (2002). Human glutathione-dependent formaldehyde
dehydrogenase. Biochemistry.

[B62] Sasnaukas K, Jomantiené R, Januska A, Lebediené E, Lebedys J, Janulaitis A (1992). Cloning and analysis of a *Candida maltosa* gene
which confers resistance to formaldehyde in *Saccharomyces
cerevisiae*. Gene.

[B63] Schwarz G (1978). Estimating the dimension of a model. Ann Statist.

[B64] Strömberg P, Höög J (2000). Human class V alcohol dehydrogenase (ADH5): A complex
transcription unit generates C-terminal multiplicity. Biochem Biophys Res Commun.

[B65] Tavaré S (1986). Some probabilistic and statistical problems in the analysis of
DNA sequences. Lectures Math Life Sci.

[B66] Tennessen JA (2008). Positive selection drives a correlation between non-synonymous /
synonymous divergence and functional divergence. Bioinformatics.

[B67] Thompson CE, Salzano FM, Norberto de Souza O, Freitas LB (2007). Sequence and structural aspects of functional diversification of
plant alcohol dehydrogenases. Gene.

[B68] Thompson CE, Fernandes CL, Norberto de Souza O, Freitas LB, Salzano FM (2010). Evaluation of the impact of functional diversification on
Poaceae, Brassicaceae, Fabaceae, and Pinaceae alcohol dehydrogenase
enzymes. J Mol Model.

[B69] Thomson JM, Gaucher EA, Burgan MF, De Kee DW, Li T, Aris JP, Benner SA (2005). Resurrecting ancestral alcohol dehydrogenases from
yeast. Nat Genet.

[B70] Wendel JF (2000). Genome evolution in polyploids. Plant Mol Biol.

[B71] Whelan S, Goldman N (2001). A general empirical model of protein evolution derived from
multiple protein families using a maximum-likelihood
approach. Mol Biol Evol.

[B72] Williamson VM, Paquin CE (1987). Homology of *Saccharomyces cerevisiae* ADH4 to an
iron-activated alcohol dehydrogenase from *Zymomonas mobilis*. Mol Gen Genet.

[B73] Wills C, Jörnvall H (1979). The two major isoenzymes of yeast alcohol
dehydrogenase. Eur J Biochem.

[B74] Yang Z (2007). PAML 4: Phylogenetic Analysis by Maximum
Likelihood. Mol Biol Evol.

[B75] Young ET, Sloan J, Miller B, Li N, van Riper K, Dombek KM (2000). Evolution of a glucose-regulated ADH gene in the genus
*Saccharomyces*. Gene.

[B76] Zgombic-Knight M, Ang HL, Foglio MH, Duester G (1995). Cloning of the mouse class IV alcohol dehydrogenase (retinol
dehydrogenase) cDNA and tissue-specific expression patterns of the murine
ADH gene family. J Biol Chem.

[B77] Zheng Y, Xu D, Gu X (2007). Functional divergence after gene duplication and
sequence-structure relationship: a case study of G-protein alpha
subunits. J Exp Zool B.

[B78] Zheng Y-W, Bey M, Liu H, Felder MR (1993). Molecular basis of the alcohol dehydrogenase-negative deer
mouse. Evidence for deletion of the gene for class I enzyme and identification
of a possible new enzyme class. J Biol Chem.

