# Supplementary material for: Molecular evolution and functional divergence of alcohol dehydrogenases in animals, fungi and plants
Source: Genet Mol Biol. 2018;41(1 Suppl 1):341–54. doi: 10.1590/1678-4685-GMB-2017-0047 (PMC5913725; doi:10.1590/1678-4685-GMB-2017-0047)
Supplement: Supplementary file 2 [file 1415-4757-GMB-41-01-2017-0047-s002.pdf]

Supplementary Material to “Molecular evolution and functional divergence of alcohol dehydrogenases in animals, fungi and plants”

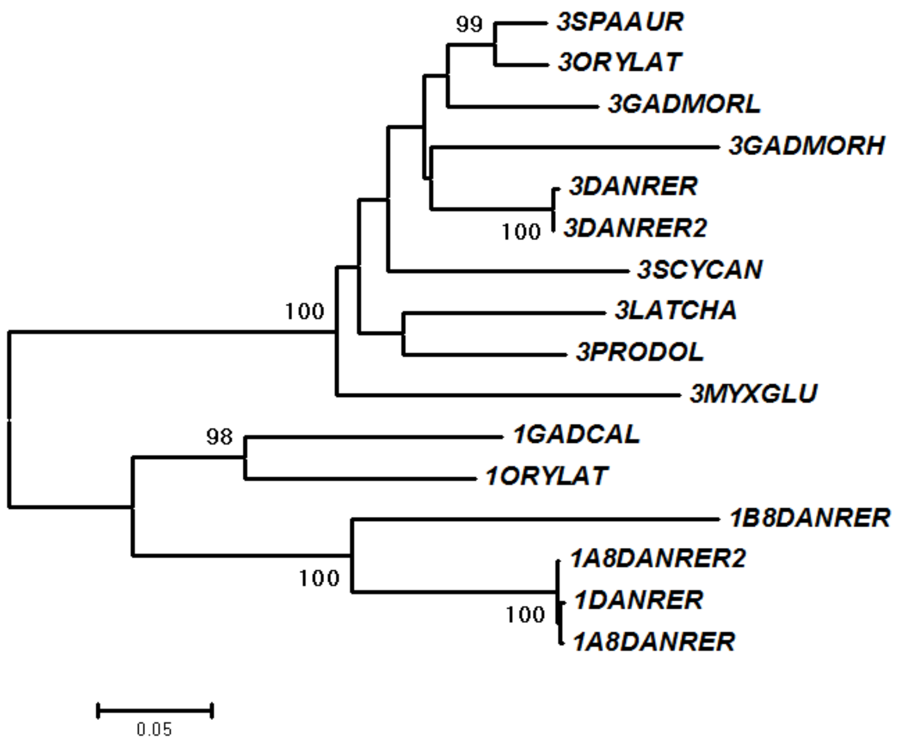

Figure S1 – Evolutionary relationships of fish ADH proteins obtained using the neighbor-joining method, Poisson-corrected amino acid distances, and pairwise deletion of gaps/missing data. Numbers representing bootstrap values higher than 80% are shown. Scale bar indicates levels of sequence divergence.
