# Supplementary material for: Molecular evolution and functional divergence of alcohol dehydrogenases in animals, fungi and plants
Source: Genet Mol Biol. 2018;41(1 Suppl 1):341–54. doi: 10.1590/1678-4685-GMB-2017-0047 (PMC5913725; doi:10.1590/1678-4685-GMB-2017-0047)
Supplement: Supplementary file 4 [file 1415-4757-GMB-41-01-2017-0047-s004.pdf]

Supplementary Material to "Molecular evolution and functional divergence of alcohol dehydrogenases in animals, fungi and plants"

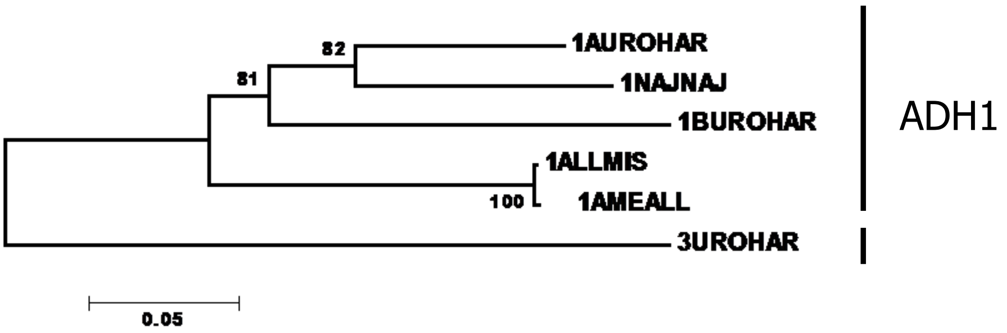

Figure S3 – Evolutionary relationships of reptilian ADH proteins obtained using the neighbor-joining method and Poisson-corrected amino acid distances. Bootstrap values higher than 80% are shown. Scale bar indicates the sequence divergence level.
